# Concerted Cultivation and Adolescent Psychopathology over Time-Mediation of Parent-Child Conflict

**DOI:** 10.3390/ijerph17249173

**Published:** 2020-12-08

**Authors:** Janet T. Y. Leung

**Affiliations:** Department of Applied Social Sciences, The Hong Kong Polytechnic University, Hong Kong; janet.leung@polyu.edu.hk

**Keywords:** concerted cultivation, adolescent psychopathology, parent–child conflict, Chinese, longitudinal study

## Abstract

Background: Concerted cultivation is a parenting strategy that parents nurture their children intensively by involving heavily in their children’s academic sphere as well as offering them different structured “enrichment” activities so that their children can succeed in the future competitive “rug rat race”. While this parenting strategy has been regarded as an effective strategy to promote child and adolescent development, it is deemed to create stress and anxiety for their children. The present study examined the relationship between concerted cultivation and adolescent psychopathology (indexed by depression and anxiety) via parent–child conflict among Chinese adolescents in Hong Kong over time. Method: A sample of 1570 young adolescents (48.5% girls, mean age at time 1 = 12.6, *SD* = 0.76) were recruited from 19 secondary schools in Hong Kong. Adolescents were invited to fill out a questionnaire that contained measures of concerted cultivation, parent–child conflict, anxiety and depression in two consecutive years. Results: Results from structural equation modeling showed that higher levels of paternal concerted cultivation were associated with higher levels of adolescent psychopathology via increased father–child conflict over time. However, maternal concerted cultivation was linked to greater mother–child conflict but reduced father-child conflict, which was associated with adolescent psychopathology. Discussion: Rather than regarding concerted cultivation as an effective parenting strategy that promotes adolescent development, the findings indicated that concerted cultivation increased adolescent psychopathology via increased parent–child conflict. The study sheds new light for family practitioners and educators in their awareness of the adverse effects of concerted cultivation and designing appropriate parent education programs for parents.

## 1. Introduction

In recent decades, globalization has changed the landscape of job markets [1], and the rise of meritocracy has heightened fierce competition among young generations [2]. To promote future competitiveness for teenagers, parents seize every opportunity to nurture their children so that they can eventually win in the competitive “rug rag race” [3]. In many East Asian countries (e.g., China, South Korea, Japan), parents are highly involved in the school performance of their children and advance their children’s talents with different enrichment programs [4,5,6]. Likewise, in an ethnographic study on exploring how middle-class families promoted the talents of their children, Lareau [7] used the term “concerted cultivation” to describe the effortful investment of parents in cultivating the talents of their children. Concerted cultivation has been regarded as an effective parenting strategy to enhance children’s and adolescents’ competencies [8].

While a majority of studies focus on examining the relationship between concerted cultivation and academic achievement of children and adolescents [9,10,11], there are fewer studies exploring the effects of concerted cultivation on adolescent mental health [12,13]. Moreover, the mechanisms through which concerted cultivation affects adolescent mental wellbeing are seldom explored. Furthermore, most studies presumed that mothers are caregivers and hence are responsible for cultivating their children’s talents, while paternal contributions to cultivating their children’s talents were ignored in the literature. Last but not least, though concerted cultivation is prominent in the Chinese culture, related studies in the Chinese communities are severely lacking. As such, the present study examined the longitudinal effects of paternal and maternal concerted cultivation on adolescent psychopathology via father–child and mother–child conflict among a sample of Chinese adolescents in Hong Kong.

### 1.1. Concerted Cultivation in the Chinese Culture

Originally, the concepts of concerted cultivation were analyzed under the contextual framework in examining different approaches of socialization strategies adopted by middle-class and working-class families [7,14]. Lareau [14] suggested that middle-class parents transmit educational advantages to their children by means of parental involvement in children’s school life, development of language patterns using a rich vocabulary that facilitates reasoning and negotiation, and provision of structural leisure activities for their children [7,10]. This “interventionist” approach to socialization strategies was proved to be effective in promoting children’s and adolescents’ cognitive skills and school performance [9,11].

In Chinese culture, concerted cultivation is regarded as a parental investment for promoting the future success of their children [15]. Chinese parents involve heavily in child education [16] and provide resources for their children to attend private tutoring and enrichment programs [5]. These socialization strategies become more appealing when facing fierce competition in schools and future job markets. Concerted cultivation has both cultural and instrumental meanings to Chinese families. According to Confucian thought, education and scholarship are intrinsic in establishing human malleability and character improvement [17], which are fundamental for the development of a “*chun-tzu*” (man of virtue or noble character) [18]. Concerted cultivation that focuses on education and talent development is important for building up the characters of children and adolescents. Instrumentally, education, and talent development help people climb up the social ladder, which is critical for future economic stability [19]. This is particularly important for Chinese families, as children’s success and achievement bring family honor [20]. Hence, Chinese parents are obligated to invest in the development and future betterment of their offspring [20,21]. They put more emphasis on the school performance of their children and foster their talents and skills so that their children can be more advantageous in educational advancement and future job security [22]. As a result, they involve heavily in their children’s schoolwork and examinations and are obsessive in searching for different talent enrichment programs for their children. However, as Chinese parents are more controlling and directive to their children [23,24], and parental position is more hierarchical than that in the Western culture [25], it is unlikely that parents may regard a language pattern of “reasoning and negotiation” as a “cultural capital” and transmit it to their children. Hence, in conceptualizing “concerted cultivation” under the Chinese culture, “parental academic involvement” and “intensive scheduling of enrichment activities” are mainly focused.

### 1.2. Relationship among Concerted Cultivation, Parent–Child Conflict and Adolescent Psychopathology

Based on the cultural reproduction theory, parents transmit their values and skills to their children via socialization practice and family investment [26]. Believing that education and talent development is crucial for their children’s cognitive development and future success, parents involve intensively in their children’s school life and sign up for different enhancement programs for their children. However, the packed schedule of structured programs may lead to pressure and exhaustion in children and adolescents [12,13,27]. Elkind [28] used the term “hurried child” to describe those children who spend long hours each day joining different adult-arranged programs and lose the opportunities to interact with peers and enjoy free play. Moreover, adolescents may experience guilt and shame when they fail to fulfill parental demands for good academic performance and well-developed talents [29,30]. In this sense, parental concerted cultivation is detrimental to children’s and adolescents’ wellbeing. From a meta-analysis of parental factors associating with adolescent depression and anxiety, parental over-involvement has a small effect size on affecting adolescent anxiety but a medium effect size on affecting adolescent depression [31]. There is a popular Chinese fable describing a farmer who stretched the shoots out in order to “help” them grow faster and taller. Eventually, the shoots died. The fable serves as a metaphor that describes the adverse effect of over-enthusiastic involvement of parents that may spoil their children.

While a majority of related studies selected children as the target sample [32,33], few studies paid attention to its impacts on early adolescents. In fact, early adolescents may react more rigorously towards concerted cultivation. In adolescence, teenagers strive for greater independence and autonomy in the individuation process [34] and request for more differentiation from their parents [35]. Concerted cultivation implies parental intrusion to adolescents’ life routine and time–space, which may generate parent–child conflict and create stress for adolescents. Based on the self-determination theory [36], the lack of autonomy granting from parents may lead to poorer relatedness between parents and adolescents, which in term may affect adolescents’ competencies and wellbeing. Hence, it is portrayed that concerted cultivation is linked to adolescent psychopathology via the generation of parent–child conflict.

In the existing literature, a high proportion of studies did not differentiate paternal and maternal contributions on concerted cultivation (e.g., [9]), or they simply presumed mothers as providers of concerted cultivation for their children [10,37]. Little is known about paternal influences on adolescent wellbeing as well as the differential effects of paternal and maternal over-involvement on adolescent mental health [38,39]. Among the few related studies, the results were equivocal. While some studies showed that maternal over-involvement and intrusion were associated with poorer mental health, greater adolescent anxiety and more avoidance of mothers [40,41], other studies showed that paternal over-involvement and control were associated with adolescent anxiety and father–child conflict [42,43]. Moreover, according to the family systems theory, one subsystem may affect the other subsystems within a family [44,45]. The parenting strategies of one parent may affect the parental quality of another parent. Adolescents who experience forceful parenting may turn to other family members for support [46]. Regarding concerted cultivation, it is possible that intensive cultivation of one parent may lead to a poorer relationship with that parent, but at the same time enhance the relationship with another parent for more support and autonomy granting. As the interrelationships among paternal and maternal concerted cultivation and father–child and mother–child conflict were not explored, this study was pioneering in examining the interrelationships.

### 1.3. Moderating Roles of Adolescent Gender, Family Intactness and Family Hardship

In this study, three moderators of demographic characteristics, adolescent gender, family intactness and family economic hardship, were examined. For adolescent gender, while a majority of the studies showed that parental over-involvement was more impactful on parent–child conflict and wellbeing for girls than boys [42,47,48], other studies showed that there was no significant difference between parental over-control and adolescent wellbeing across adolescent gender [43].

For family intactness, adolescents from single-mother families are more indebted to their mothers for their sacrifice and investment and strive for excellence in order to reciprocate their mothers [49]. Hence, it is reasonable to hypothesize that the relationship between maternal concerted cultivation and parent–child conflict would be weaker in non-intact families than in intact families.

Though concerted cultivation has been regarded as a middle-class socialization strategy [7,10], there is no adequate evidence showing that the relationship between concerted cultivation and adolescent wellbeing is different across socioeconomic status. On the contrary, economically disadvantaged adolescents may express greater gratitude towards their parents for their investment despite the scarce resources their families possess [50]. The resources and talent development may further bring hope to those adolescents. As research on the moderating roles of adolescent gender, family intactness and family economic hardship on the relationship between concerted cultivation and adolescent wellbeing is severely lacking, the examination of moderating effects of these demographic characteristics is considered exploratory in the present study.

### 1.4. The Current Study

The study examines the relationships among perceived concerted cultivation (indexed by “parental academic involvement” and “intensive scheduling of enrichment activities”), parent–child conflict and adolescent psychopathology (indexed by anxiety and depression) among Chinese adolescents in Hong Kong. There are several hypotheses:

**Hypothesis** **1** **(H1).** *Father-child conflict mediates the positive relationship between paternal concerted cultivation and adolescent psychopathology.*

**Hypothesis** **2** **(H2).** *Mother-child conflict mediates the positive relationship between maternal concerted cultivation and adolescent psychopathology.*

**Hypothesis** **3** **(H3).** *Higher paternal concerted cultivation is associated with decreased mother–child conflict over time.*

**Hypothesis** **4** **(H4).** *Higher maternal concerted cultivation is associated with decreased father–child conflict over time.*

**Hypotheses** **5** **(H5).** *Higher paternal concerted cultivation is associated with greater father-child conflict for adolescent girls***(H5a)**, *non-intact families***(H5b)***and families with higher income***(H5c)**.

**Hypotheses** **6** **(H6).** *Higher maternal concerted cultivation is associated with greater mother-child conflict for adolescent girls***(H6a),***non-intact families***(H6b)***and families with higher income***(H6c)**.

## 2. Materials and Method

### 2.1. Participants and Procedures

Nineteen secondary schools across Hong Kong joined the current study. At time 1 (T1), 1735 students of secondary one (grade 7) participated in the study. They were invited to fill out the questionnaire again in the consecutive year at time 2 (T2). After matching, 1570 respondents completed the questionnaires at the two time points, with an attrition rate of 9.5%. In this study, 1570 sets of T1 and T2 questionnaires were analyzed.

Among the 1570 respondents at T1, 803 (51.1%) were boys and 762 (48.5%) were girls (5 persons did not respond). The mean age was 12.61 (SD = 0.76). While 1185 (75.5%) respondents came from intact families, 321 (20.5%) respondents came from non-intact families, including 131 (8.3%), 111 (7.1%), 48 (3.1%) and 31 (2.0%) adolescents growing up in remarried, divorced, separated and widowed families, respectively (n = 27 (1.7%) reported others; n = 37 (2.4%) did not respond). There were 442 (28.2%) respondents living in poverty, whose families were recipients of either comprehensive social security assistance (CSSA) or full textbook allowance (FTBA) from the government (n = 252, 16.1% did not respond).

Prior to data collection, informed written consent from parents and adolescents were sought. Data collection was performed in the class lessons by the researcher and/or trained research assistants. The students were requested to fill out a questionnaire that contained the measures of perceived paternal and maternal concerted cultivation, father-child and mother-child conflict, anxiety, depression and demographic characteristics in a self-administered format. For those students who did not join the study, they did their home assignments in class. The students used appropriately 20 min to complete the questionnaires. Identical procedures were repeated in the next consecutive year. To safeguard the ethical requirement of human subjects, ethical approval was obtained from the Human Subjects Ethics Subcommittee of an internationally recognized university.

### 2.2. Instruments

#### 2.2.1. Perceived Concerted Cultivation

*Perceived Paternal and Maternal Concerted Cultivation Scale (PCC and MCC).* Based on the literature of concerted cultivation [7,9] as well as the Chinese overparenting behavior [51], the 10-item PCC and MCC were developed. The measurements possess two dimensions, parental academic involvement (5 items) and intensive scheduling of enrichment activities (5 items). An item of “parental academic involvement” reads “My father/mother involves intensively in preparing me for examination,” and that of “intensive scheduling enrichment activities” reads “My father/mother fills out my schedule with numerous activities”. Each item is rated on a 6-point Likert scale (1 = “strongly disagree” and 6 = “strongly agree”). The scale is attached in Appendix A. Higher mean scores of PCC and MCC indicate higher levels of paternal and maternal intensive cultivation perceived by adolescents, respectively. Both PCC and MCC indicated good reliability in the study (PCC and MCC: α at T1 = 0.90 and 0.89, inter-item correlations at T1 = 0.47 and 0.44).

#### 2.2.2. Parent-Child Conflict

*Father-Adolescent Conflict and Mother-Adolescent Conflict Scales (FAC and MAC).* Shek [52] translated the conflict behavior questionnaire [53] into the Chinese version of FAC and MAC, respectively. The 3-item short forms of FAC and MAC were used, which possessed good psychometric properties in previous studies [42]. The three items were “I always have conflict with my father/mother”, “I and my father/mother always quarrel with each other”, and “My father/mother and I always criticize or pick on each other”. Both FAC and MAC are assessed on a 6-point Likert scale from 1 = “strongly disagree” to 6 = “strongly agree”. Higher mean scores of FAC and MAC indicate higher levels of father-child and mother-child conflict, respectively. Both FAC and MAC indicated good internal consistency in T1 and T2 (FAC: α at T1 and T2 = 0.89 and 0.91, inter-item correlations at T1 and T2 = 0.73 and 0.77; MAC: α at T1 and T2 = 0.90 and 0.90, inter-item correlations at T1 and T2 = 0.74 and 0.74).

#### 2.2.3. Adolescent Psychopathology

*The Chinese Hospital Anxiety and Depression Scale (HADS-C)*. Leung et al. [54] translated the Hospital Anxiety and Depression Scale (HADS; [55]) into the Chinese version of HADS (HADS-C) and showed acceptable psychometric properties in a validation study [56]. There are two dimensions of HADS-C: A 7-item anxiety subscale and a 7-item depression subscale. A sample item of anxiety subscale reads “I feel tense or ‘wound up’”, and that of depression subscale reads “I have lost interest in my appearance” (for the full scale, please refer to [55], pp. 368–370). Each item is assessed on a 4-point Likert scale from “0 = not at all” to “3 = most of the time”. Higher mean scores of anxiety subscale and depression subscale indicate higher levels of anxiety and depression, respectively. Both subscales showed acceptable reliability of both time points (anxiety subscale: α at T1 and T2 = 0.75 and 0.76, inter-item correlations at T1 and T2 = 0.30 and 0.33; depression subscale: α at T1 and T2 = 0.69 and 0.67, inter-item correlations at T1 and T2 = 0.24 and 0.23).

#### 2.2.4. Demographic Characteristics

Three demographic characteristics, adolescent gender (boys = 0; girls = 1), family structure (non-intact group [including remarried, divorced, separated and widowed families] = 0; intact families = 1) and economic hardship (non-poor group = 0; poor group [families receiving government financial assistance] = 1), were collected during data collection.

### 2.3. Data Analysis

Correlational analyses were performed to examine the bivariate relationships among the studied variables. Structural equation modeling (SEM) using the software program of AMOS 26.0 was used in the analyses. The procedures suggested by Cole and Maxwell [57] were adopted to test the mediation model. First, confirmatory factor analysis (CFA) was conducted among perceived paternal and maternal concerted cultivation, father-child and mother-child conflict, and adolescent psychopathology. Every latent variable was allowed to relate to every other latent variable. The indicators of CFI > 0.90 and RMSEA < 0.06 showed a good fit of the model, while CFI > 0.90 and RMSEA < 0.08 indicated an acceptable fit [58]. In case the measurement model showed an acceptable fit of the data, and the observed variables corresponded to relevant latent variables, the factor structures of all latent variables were confirmed. As concerted cultivation contains two dimensions of “parental academic involvement” and “intensive scheduling of enrichment activities”, the hierarchical factor structure of paternal and maternal concerted cultivation was tested. Similarly, the hierarchical factor structure of adolescent psychopathology was also tested. Second, factor invariance of all variables across time was tested. Configural factorial invariance (i.e., a same pattern of fixed and free factor loadings of latent variables across time), weak factorial invariance (i.e., invariances in factor loadings of latent variables across time), strong factorial invariance (i.e., invariances in factor loadings and intercepts of latent variables across time), and strict factorial invariance (invariances in factor loadings, intercepts and unique factor variables of latent variables across time) were tested [59]. The CFI value of the subsequent invariance model was compared with the preceding invariance model, with ΔCFI < 0.01 as an indicator of measurement invariance across time [60]. When both confirmatory factor structure and measurement invariances among the variables were confirmed, the structural model testing the mediation effects of father-child and mother-child conflict on the relationships among perceived paternal and maternal concerted cultivation and adolescent psychopathology was performed. A bootstrapping mediation test [61] with 5000 bootstrapped re-samples was conducted to examine the significance of indirect effects. An indirect effect was confirmed when a “zero” value was not identified between the upper and lower bounds of bias-corrected 95% confidence intervals [61]. The effect size of the indirect effect was taken as the ratio of indirect effect on total effect [62]. If the signs of direct and indirect effects were opposite, absolute values of the effects were considered before calculating the ratio [63].

Multiple group analyses were conducted to examine whether adolescent gender, family intactness and family economic hardship would moderate the relationships among concerted cultivation, parent-child conflict and adolescent psychopathology. The goodness-of-fit indices of CFI > 0.90 and RMSEA < 0.08 was used to evaluate the unconstrained model [58]. Regarding adolescent gender, the regression coefficient of each structural path was constrained to be equal between male and female groups. When a difference in chi-squared values between the imposed equality constrained model and the unconstrained model was found, a moderating effect was suggested. Identical procedures were performed in assessing whether family intactness and family economic hardship would be moderators of the indirect effects.

## 3. Results

### 3.1. Preliminary Analyses

The missingness of the variables was examined in the study. The amount (ranged from 0.1% and 5.7%) and pattern analyses of missing values supported that the data were missing completely at random data (MCAR) [64]. Full information maximum likelihood (FIML) estimation was used to handle missing data, as studies showed that this approach was less biased when compared with other approaches to dealing with missing data [65]. The maximum-likelihood method was adopted in the analyses, with the skewness and kurtosis values of all variables were smaller than 2 and 7, respectively [66] (see Table 1).

Correlational analyses showed that while paternal concerted cultivation at T1 was positively related to father-child and mother-child conflict at T1 and T2, and adolescent anxiety at T1, maternal concerted cultivation at T1 were positively related to father-child conflict, mother-child conflict, adolescent anxiety and depression at T1 and T2. Moreover, boys perceived higher levels of paternal and maternal concerted cultivation at T1 and more depression at T2 than did girls. For family intactness, adolescents growing up in intact families showed higher levels of paternal concerted cultivation at T1 and less depression at T2 than did those raising up in non-intact families. No significant associations between family economic hardship and other studied variables were found (see Table 2).

Prior to the examination of the main effects of concerted cultivation and mediational effect via parent-child conflict, the linear mixed model analyses using SPSS was performed to examine whether there were random effects in adolescent anxiety and depression at T2 between schools (i.e., school effects). The analyses showed that there was significant heterogeneity in adolescent anxiety at the within-school level (Estimate = 0.29, SE = 0.01, *p* < 0.001), but was not significant at the between-school level (Estimate = 0.002, SE = 0.00, *p* > 0.05). The ICC was 0.007 (0.002/[0.002 + 0.29]), suggesting that only 0.7% of the total anxiety score variability was due to differences between schools. Similarly, significant heterogeneity in adolescent depression was identified at the within-school level (Estimate = 0.26, SE = 0.01, *p* < 0.001), but was non-significant at the between-school level (Estimate = 0.004, SE = 0.00, *p* > 0.05). The ICC value was 0.015 (0.002/[0.002 + 0.29]), suggesting that 1.5% of total depression score variability was due to differences between schools. As both ICC values of adolescent anxiety and depression at T2 were smaller than 0.05, the clustering of observations within schools was minimal [67].

### 3.2. The Measurement Model

To test the measurement model of perceived paternal and maternal concerted cultivation, father-child and mother-child conflict, and adolescent psychopathology, confirmatory factor analysis was performed. By allowing every latent variable to associate freely with every other latent variable, the measurement model (M1) showed a good fit of the data, with CFI = 0.909 (CFI > 0.90; [58]), and RMSEA = 0.038 (RMSEA < 0.06; [58]) (Table 3). Paternal concerted cultivation showed a hierarchical factor structure of “paternal academic involvement” and “intensive paternal scheduling of enrichment activities”, with factor loadings of 0.73 and 0.97, respectively. Similarly, maternal concerted cultivation showed a hierarchical factor structure of “maternal academic involvement” and “intensive, maternal scheduling of enrichment activities”, with factor loadings of 0.72 and 0.92, respectively (see Table 4). Adolescent psychopathology also showed a hierarchical factor structure of anxiety and depression, with factor loadings of 0.81 and 0.82 at T1, and 0.76 and 0.81 at T2, respectively (Table 4). Except factor loadings of Item 4 (i.e., “I feel as if I am slowed down”) and Item 5 (i.e., “I have lost interest in my appearance”) of “adolescent depression” that were lower than 0.40 at T1 and T2, all items had factor loadings greater than 0.40. All items corresponded to relevant variables of paternal and concerted cultivation at T1, and father-child conflict, mother-child conflict and adolescent psychopathology at T1 and T2, respectively. In general, the measurements showed good factorial validity.

Measurement invariance tests showed that all studied variables showed weak factorial invariance when imposing equality constraints in factor loadings of latent variables across time (M2a) and comparing M2a with the unconstrained model (i.e., M1) (Δ*x*^2^ = 7.251; *p* > 0.05, ΔCFI = 0.000; Table 3). Strong factorial invariance (ΔCFI = 0.002) and strict factorial invariance (ΔCFI = 0.001) of studied variables across time were also identified ([59]; Table 3). Hence, the factor structures of studied variables were invariant across time.

### 3.3. The Structural Model

**Figure 1:**
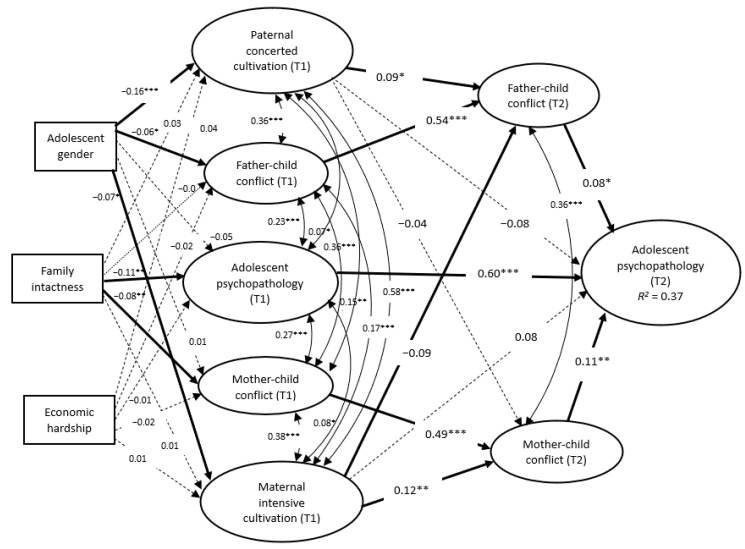
The final model of relationship among concerted cultivation, parent-child conflict and adolescent psychopathology. * *p* < 0.05, ** *p* < 0.01, *** *p* < 0.001. Note 1: Solid lines represent significant structural paths. Dotted lines represent non-significant structural paths.

When the measurement model was confirmed, the mediation model (M3) was tested. M3 indicated a good fit of the data, with CFI = 0.904 and RMSEA = 0.037 (CFI > 0.90 and RMSEA < 0.06; (58)) (Table 3). When controlling for father-child conflict and adolescent psychopathology at time 1, father-child conflict at T2 mediated the association of paternal concerted cultivation at T1 with adolescent psychopathology at T2 (standardized indirect effect = 0.05; *p* < 0.05; 95% CI = (0.000; 0.009)), with an effect size of 0.14. Mother-child conflict at T2 also mediated the association of maternal concerted cultivation at T1 with adolescent psychopathology at T2, after controlling for mother-child conflict and adolescent psychopathology at T1 (standard indirect effect = 0.013; *p* < 0.01; 95% CI = (0.001; 0.013)), with the effect size of 0.13. However, maternal concerted cultivation at T1 was negatively related to father-child conflict at T2 (*β* = −0.08, *p* < 0.05), which was further linked to adolescent psychopathology (standard indirect effect = −0.007; *p* < 0.05; 95% CI = (−0.007; 0.000)), with the effect size of 0.07. The direct effects of paternal and maternal concerted cultivation at T1 on adolescent psychopathology at T2 were non-significant, with *β* = −0.04 and 0.08 (*p* > 0.05), respectively (see Figure 1).

### 3.4. Moderating Effects of Adolescent Gender, Family Intactness and Family Economic Hardship

To examine whether adolescent gender would moderate the indirect effects of parent-child conflict on the relationship between concerted cultivation and adolescent psychopathology over time, multiple group analysis was performed. There was a difference between unconstrained (M4a) and constrained (M4b) models between boys and girls, with Δ*x*^2^ = 364.834 (*p* < 0.001) (Table 5). When equality constraint of each structural path contributing to the indirect effects was imposed between boys and girls groups, the models of imposed constraints (M4c to M4j) did not show significant differences in chi-squared values when compared with the unconstrained model (M4a) (Table 5), indicating that there were gender invariances on the indirect effects of parent-child relationship in the association between concerted cultivation and adolescent psychopathology. Similar results were indicated in assessing family intactness as a moderator (M5a to M5j; Table 5). For family economic hardship, there was invariance between adolescents living in poverty and those who were not, with Δ*x*^2^ = 153.010 (*p* > 0.05) between unconstrained (M6a) and constrained (M6b) models (Table 5). In summary, adolescent gender, family intactness and family economic hardship were not moderators that moderated the indirect effects between concerted cultivation and adolescent psychopathology via parent-child conflict.

## 4. Discussion

The present study examined the relationship between perceived concerted cultivation and adolescent psychopathology via parent-child conflict in a longitudinal study of a sample of Chinese adolescents in Hong Kong. The results indicated that perceived paternal concerted cultivation was positively associated with father-child conflict, respectively, which were further linked to adolescent anxiety and depression over time. Similarly, perceived maternal concerted cultivation were positively associated with adolescent anxiety and depression via mother-child conflict over time. Echoing the self-determination theory [36], concerted cultivation is perceived as a parental intrusion to their private life of adolescents, and intensively scheduled enrichment programs may oppress adolescents from their engagement with peers and enjoyment of their free time, which may lead to parent-child conflict. Parent-child conflict further worsen adolescent mental health when adolescents experience disagreement with their parents [52]. Interestingly, maternal concerted cultivation was negatively related to father-child conflict over time. One possibility is that when an adolescent has a conflict with his/her mother due to her intensive cultivation, he/she may turn to his/her father for gaining support to further negotiate with his/her mother, which helps to enhance father-child relationship. According to the family systems theory [35], coalitions will be formed between the father and the adolescent when both disagree with the mother’s parenting behavior [46]. However, paternal concerted cultivation did not show a significant association with mother-child conflict. Since mothers are regarded as the caregivers in the families who provide nurturance for their children, their intrusions are more apparent than the fathers’. The adolescents may turn to their father for support; particularly, fathers are more influential in the Chinese family systems where patriarchal hierarchy is stressed [68], which may reduce father-child conflict. As research on the impacts of concerted cultivation on family dynamics is almost non-existent, future studies in this area are suggested.

Furthermore, though it was portrayed that the adolescents growing up in non-intact families and those in economic hardship would be more indebted to their parents’ investment and sacrifice through concerted cultivation, which would reduce parent-child conflict, the results did not show the moderating effects of family intactness and family economic hardship in the relationship between concerted cultivation and parent-child conflict over time. Regardless of family structure and economic hardship, adolescents generally feel the pressure from the intrusion of parents on their academic performance and extra-curricular activities, which may generate parent-child conflict. In view of inconsistent findings of adolescent gender as a moderator [43,48], the study provides additional evidence that adolescent gender did not moderate the direct and indirect relationship between concerted cultivation and adolescent psychopathology via parent-child conflict. Both adolescent boys and girls may feel frustrated with parent-child conflict generated by intensive parental cultivation.

There are several theoretical and practical implications of the study. Theoretically, concerted cultivation was mostly studied in Western society [9,10,11], while related research conducted in the Chinese communities was severely lacking. Though Chinese parents are obligated to sacrifice and invest for their children [69], they are more controlling in parenting [23] and may not transmit a language pattern of negotiation and reasoning to their children, as what was found in the Western context [7]. However, they strongly value their children’s academic achievement and talent development [70,71]. As conceptualization of concerted cultivation is culturally specific [26,72], Chinese concerted cultivation may be different from the Western concerted cultivation. This study conceptualized Chinese concerted cultivation as parental strategies that cultivate their children’s talents and competencies via intensive parental involvement in their children’s school performance and schedule of enrichment activities for them, which brings important addition to the existing literature.

While concerted cultivation is deemed as an effective strategic approach of parents in promoting children’s and adolescents’ cognitive competencies [9,11], the negative effects of this parental intervention approach on child and adolescent wellbeing are seldom explored, particularly at the stage of adolescence. The findings suggested that concerted cultivation was positively associated with adolescent anxiety and depression via parent-child conflict. There has been a general belief that parents ought to prepare a ‘winning’ start for their children [73]. Hence, parents become obsessive to search for different tutorials and enrichment programs for their children [5,16]. However, the study showed that too much parental involvement might hamper adolescent wellbeing, as adolescents perceived intensive cultivation as an intrusion to their private life, which may lead to parent-child conflict. The study sheds new light on examining the mechanisms through which parent-child conflict mediates the effects of concerted cultivation on wellbeing in Chinese adolescents, which helps in the development of the Chinese model on family socialization.

Moreover, the study made a differentiation between paternal and maternal concerted cultivation and examined each effect on adolescent psychopathology via father-child and mother-child conflict, which brings conceptual advance to the related study. The findings showed an indirect effect of father-child conflict in the relationship between paternal concerted cultivation and adolescent psychopathology. However, maternal concerted cultivation increased mother-child conflict but reduced father-child conflict over time. The findings showed how parent gender made impacts on socialization strategies and parent-child conflict, which is crucial for future investigations of family interactions that may affect adolescent wellbeing.

Practically, family practitioners and youth counselors may need to be sensitive to the potential impacts of intensive cultivation that may hamper parent-child relationship and adolescent wellbeing. With the understanding of parents’ good will in nurturing their children, family practitioners and youth counselors can clarify with parents on the “what” and “how much” of cultivation should be exercised for their adolescent children. Moreover, as maternal concerted cultivation may affect both father-child and mother-child relationships, it is necessary to observe the family dynamics when concerted cultivation is practiced.

There are some limitations to the study. First, the study involved two-wave longitudinal data with an interval of one year in examining the relationships among concerted cultivation, parent-child conflict and adolescent psychopathology over time. Though early adolescents are more critical and rebellious towards the forceful arrangement of their parents [74], a two-wave longitudinal research design may not be adequate to fully capture the effects. A longitudinal study of multiple years is suggested in future studies. Furthermore, though SEM was adopted in the analyses, which has the strengths to control measurement errors, confirm tested models by assessing their goodness of fit and estimate the direct and indirect effects among the variables [75], it has the inherent limitation to draw causal inference among the studied variables.

Another limitation lies in the recruitment of a single source of informants (i.e., adolescents) in this study. Though it is reasonable for adolescents to rate their perceptions of concerted cultivation because they are “receivers” of this parenting strategy [76] and their subjective perceptions are critical in influencing their mental health [77], it is advised to collect the data from different sources (including fathers and mothers) so as to improve the generalizability of the results.

Furthermore, rather than using objective parameters of concerted cultivation (e.g., number of enrichment programs enrolled, time spent on enrichment activities), the study used subjective adolescents’ perceptions of concerted cultivation in the operationalization of the construct. Though it is justified that subjective outcome parameters can give us a clearer understanding of the quality of concerted cultivation experienced by adolescents, it is also important to examine how the intensity of parental cultivation may affect adolescent wellbeing [78]. It is recommended that the objective parameters of concerted cultivation can be added in future research. In addition, the study showed that maternal concerted cultivation was positively associated with mother-child conflict but negatively related to father-child conflict. Marital quality may matter in accounting for the dynamics, as the marital subsystem affects father-child and mother-child subsystems [45,79,80]. However, information on marital quality was not included in the present study. Hence, it is suggested that marital quality be added as a moderator of the relationships in future studies.

Another limitation is that adolescents may not know the household income of the family. Hence, “recipient of public assistance” was used as the criterion for economically disadvantaged families. Invariance was found between families with and without economic hardship. However, studies from Western countries showed that concerted cultivation is a middle-class phenomenon that parents have more resources in nurturing their children. Hence, there is a need to replicate the studies among middle-class adolescents and see whether there would be a difference between adolescents growing up in middle-class and working-class families. Last but not least, the research was conducted in a sample of Chinese adolescents in Hong Kong. As Hong Kong is a former British colony and is greatly influenced by the Western culture, there is a need to replicate this study in other Chinese communities (e.g., China, Taiwan, American Chinese and European Chinese).

## 5. Conclusions

Despite the good intention of parents in enhancing educational and developmental advantages for their children through concerted cultivation, adolescents displayed more anxiety and depression when they experienced higher levels of paternal and maternal concerted cultivation over time. Moreover, parent-child conflict mediated the relationship between concerted cultivation and adolescent psychopathology. Feeling intruded by their parents in arranging different enrichment programs and emphasizing too much on their school performance, adolescents have a conflict with their parents, which in turn affects their wellbeing. This study brings new angles on examining the effects of concerted cultivation on parent-child conflict and wellbeing of Chinese early adolescents, which calls for a balance between autonomy granting and parental involvement in nurturing adolescents.

## Figures and Tables

**Table 1:** Descriptive statistics.

Time	Variables	Range	Mean	SD	Skewness	Kurtosis	Cronbach’s Alpha
T1	Paternal concerted cultivation	1–6	2.30	0.95	0.74	0.40	0.90
	Paternal academic involvement	1–6	2.73	1.19	0.42	−0.53	0.88
	Paternal provision of structured activities	1–6	1.88	0.93	1.28	1.78	0.85
	Maternal concerted cultivation	1–6	3.07	1.06	0.16	−0.35	0.89
	Maternal academic involvement	1–6	3.63	1.28	-0.13	−0.64	0.87
	Maternal provision of structured activities	1–6	2.51	1.11	0.64	0.02	0.83
	Father-child conflict	1–6	2.74	1.38	0.58	−0.40	0.89
	Mother-child conflict	1–6	3.00	1.40	0.34	−0.66	0.90
	Adolescent psychopathology	1–4	2.10	0.46	0.35	0.23	0.80
	Anxiety	1–4	2.20	0.54	0.28	0.16	0.75
	Depression	1–4	2.01	0.54	0.42	0.04	0.69
T2	Father-child conflict	1–6	2.90	1.37	0.43	−0.55	0.91
	Mother-child conflict	1–6	3.16	1.34	0.25	−0.57	0.90
	Adolescent psychopathology	1–4	2.10	0.46	0.35	0.24	0.81
	Anxiety	1–4	2.18	0.54	0.34	0.26	0.76
	Depression	1–4	2.03	0.51	0.45	−0.01	0.67

**Table 2:** Correlations of the measuring variables.

	1	2	3	4	5	6	7	8	9	10	11	12	13	14	15	16	17	18
Paternal concerted cultivation (T1)	1.00																	
2. Paternal academic involvement (T1)	0.92 ***	1.00																
3. Paternal provision of structured activities (T1)	0.86 ***	0.59 ***	1.00															
4. Maternal concerted cultivation (T1)	0.42 ***	0.35 ***	0.40 ***	1.00														
5. Maternal academic involvement (T1)	0.33 ***	0.35 ***	0.24 ***	0.90 ***	1.00													
6. Maternal provision of structured activities (T1)	0.41 ***	0.26 ***	0.50 ***	0.87 ***	0.56 ***	1.00												
7. Father-child conflict (T1)	0.31 ***	0.24 ***	0.32 ***	0.15 ***	0.08**	0.20 ***	1.00											
8. Mother-child conflict (T1)	0.13 ***	0.09 **	0.15 ***	0.35 ***	0.27 ***	0.35 ***	0.35 ***	1.00										
9. Adolescent psychopathology (T1)	0.09 ***	0.02	0.15 ***	0.14 ***	0.05	0.21 ***	0.22 ***	0.28 ***	1.00									
10. Anxiety (T1)	0.11 ***	0.07 *	0.14 ***	0.16 ***	0.08 **	0.20 ***	0.21 ***	0.26 ***	0.87 ***	1.00								
11. Depression (T1)	0.05	-0.03	0.13 ***	0.08 ***	-0.00	0.16 ***	0.17 ***	0.23 ***	0.86 ***	0.50 ***	1.00							
12. Father-child conflict (T2)	0.22 ***	0.20 ***	0.20 ***	0.06 *	0.02	0.09 ***	0.53 ***	0.18 ***	0.18 ***	0.17 ***	0.14 ***	1.00						
13. Mother-child conflict (T2)	0.10 ***	0.08 ***	0.11 ***	0.27 ***	0.23 ***	0.24 ***	0.22 ***	0.49 ***	0.21 ***	0.20 ***	0.17 ***	0.36 ***	1.00					
14. Adolescent psychopathology (T2)	0.04	-0.00	0.09 ***	0.13 ***	0.06 *	0.17 ***	0.13 ***	0.19 ***	0.57 ***	0.50 ***	0.49 ***	0.20 ***	0.25 ***	1.00				
15. Anxiety (T2)	0.04	0.00	0.07 **	0.13 ***	0.07 **	0.16 ***	0.14 ***	0.19 ***	0.49 ***	0.52 ***	0.34 ***	0.21 ***	0.25 ***	0.88 ***	1.00			
16. Depression (T2)	0.04	−0.01	0.09 **	0.09 ***	0.04	0.14 ***	0.08 **	0.15 ***	0.50 ***	0.35 ***	0.52 ***	0.14 ***	0.18 ***	0.86 ***	0.51 ***	1.00		
17. Adolescent gender	−0.15 ***	−0.13 ***	−0.13 ***	−0.06 *	−0.06 *	−0.04	−0.04	0.02	−0.04	−0.01	−0.07**	0.02	0.02	−0.07 **	−0.03	−0.10 ***	1.00	
18. Family structure (T1)	0.05*	0.07*	0.03	0.00	0.01	0.00	−0.01	−0.09 **	−0.10 ***	−0.05	−0.00	−0.03	−0.03	−0.06 *	−0.04	−0.06 *	−0.04	1.00
19. Economic hardship (T1)	0.02	0.01	0.02	0.01	0.01	0.01	0.01	−0.02	−0.03	−0.03	0.01	−0.04	−0.04	−0.01	0.02	−0.03	−0.05	0.16 ***

* *p* < 0.05, ** *p* < 0.01, *** *p* < 0.001.

**Table 3:** Goodness-of-fit indicators of the measurement model, structural model and invariant test model by adolescent gender, family intactness and family economic hardship.

Description	Model	*x* ^2^	*df*	CFI	RMSEA	Model Comparison	Δ*x*^2^	Δ*df*	ΔCFI
Measurement model: confirmatory factor analysis (CFA)	M1	5419.154 ***	1660	0.909	.038				
Measurement model: measurement invariance between time 1 and time 2									
Weak Invariance	M2a	5442.969 ***	1676	0.909	0.038	M2a and M1	23.815	16	0.000
Strong Invariance	M2b	5513.624 ***	1696	0.907	0.038	M2b and M2a	70.654 ***	20	0.002
Strict Invariance	M2c	5607.317 ***	1716	0.906	0.038	M2c and M2b	93.694 ***	20	0.001
Structural model:									
Mediation of parent-child conflict on the relationship between concerted cultivation and adolescent psychopathology	M3	5831.021 ***	1830	0.904	0.037				
Invariant test model by adolescent gender									
Unconstrained model	M4a	7704.398 ***	3546	0.900	0.027				
Constrained model	M4b	8069.232 ***	3675	0.894	0.028	M4b and M4a	364.834 ***	129	0.006
Equality constraint: Paternal concerted cultivation (T1) → Adolescent psychopathology (T2)	M4c	7705.027 ***	3547	0.900	0.027	M4c and M4a	0.629	1	0.000
Equality constraint: Maternal concerted cultivation (T1) → Adolescent psychopathology (T2)	M4d	7704.658 ***	3547	0.900	0.027	M4d and M4a	0.260	1	0.000
Equality constraint: Paternal concerted cultivation (T1) → Father-child conflict (T2)	M4e	7707.627 ***	3547	0.900	0.027	M4e and M4a	3.229	1	0.000
Equality constraint: Paternal concerted cultivation (T1) → Mother-child conflict (T2)	M4f	7707.778 ***	3547	0.900	0.027	M4f and M4a	3.380	1	0.000
Equality constraint: Maternal concerted cultivation (T1) → Father-child conflict (T2)	M4g	7704.961 ***	3547	0.900	0.027	M4g and M4a	0.563	1	0.000
Equality constraint: Maternal concerted cultivation (T1) → Mother-child conflict (T2)	M4h	7706.657 ***	3547	0.900	0.027	M4h and M4a	2.259	1	0.000
Equality constraint: Father-child conflict (T2) → Adolescent psychopathology (T2)	M4i	7705.212 ***	3547	0.900	0.027	M4i and M4a	0.814	1	0.000
Equality constraint: Mother-child conflict (T2) → Adolescent psychopathology (T2)	M4j	7704.854 ***	3547	0.900	0.027	M4j and M4a	0.455	1	0.000

*** *p* < 0.001.

**Table 4:** Factor loadings of the studied variables.

Variables	Factor Loading			
	Time 1		Time 2	
	1st Order	2nd Order	1st Order	2nd Order
Paternal concerted cultivation				
Paternal academic involvement		0.73		
Item 1	0.65			
Item 2	0.77			
Item 3	0.75			
Item 4	0.83			
Item 5	0.79			
Paternal provision of structured activities		0.97		
Item 1	0.79			
Item 2	0.75			
Item 3	0.73			
Item 4	0.78			
Item 5	0.64			
Maternal concerted cultivation				
Maternal academic involvement		0.72		
Item 1	0.67			
Item 2	0.79			
Item 3	0.72			
Item 4	0.84			
Item 5	0.79			
Maternal provision of structured activities		0.92		
Item 1	0.77			
Item 2	0.65			
Item 3	0.73			
Item 4	0.79			
Item 5	0.59			
Father-child conflict				
Item 1	0.83		0.86	
Item 2	0.95		0.95	
Item 3	0.79		0.83	
Mother-child conflict				
Item 1	0.86		0.86	
Item 2	0.96		0.96	
Item 3	0.78		0.77	
Adolescent pathology				
Anxiety		0.81		0.76
Item 1	0.58		0.65	
Item 2	0.61		0.66	
Item 3	0.63		0.66	
Item 4	0.43		0.47	
Item 5	0.53		0.48	
Item 6	0.40		0.40	
Item 7	0.64		0.64	
Depression		0.82		0.81
Item 1	0.49		0.51	
Item 2	0.56		0.59	
Item 3	0.60		0.58	
Item 4	0.34		0.30	
Item 5	0.37		0.31	
Item 6	0.58		0.56	
Item 7	0.46		0.49	

**Table 5:** Goodness-of-fit indicators of the measurement model, structural model and invariant test model by adolescent gender, family intactness and family economic hardship (Cont’d).

Description	Model	*x* ^2^	*df*	CFI	RMSEA	Model Comparison	Δ*x*^2^	Δ*df*	ΔCFI
Invariant test model by family intactness									
Unconstrained model	M5a	7869.717 ***	3598	0.893	0.028				
Constrained model	M5b	8043.332 ***	3677	0.892	0.028	M5b and M5a	173.615 **	129	0.001
Equality constraint: Paternal concerted cultivation (T1) → Adolescent psychopathology (T2)	M5c	7870.356 ***	3549	0.893	0.028	M5c and M5a	0.639	1	0.000
Equality constraint: Maternal concerted cultivation (T1) → Adolescent psychopathology (T2)	M5d	7869.773 ***	3549	0.893	0.028	M5d and M5a	0.057	1	0.000
Equality constraint: Paternal concerted cultivation (T1) → Father-child conflict (T2)	M5e	7869.920 ***	3549	0.893	0.028	M5e and M5a	0.203	1	0.000
Equality constraint: Paternal concerted cultivation (T1) → Mother-child conflict (T2)	M5f	7869.844 ***	3549	0.893	0.028	M5f and M5a	0.128	1	0.000
Equality constraint: Maternal concerted cultivation (T1) → Father-child conflict (T2)	M5g	7869.885 ***	3549	0.893	0.028	M5g and M5a	0.169	1	0.000
Equality constraint: Maternal concerted cultivation (T1) → Mother-child conflict (T2)	M5h	7869.854 ***	3549	0.893	0.028	M5h and M5a	0.137	1	0.000
Equality constraint: Father-child conflict (T2) → Adolescent psychopathology (T2)	M5i	7869.816 ***	3549	0.893	0.028	M5i and M5a	0.100	1	0.000
Equality constraint: Mother-child conflict (T2) → Adolescent psychopathology (T2)	M5j	7869.733 ***	3549	0.893	0.028	M5j and M5a	0.016	1	0.000
Invariant test model by economic hardship									
Unconstrained model	M6a	7360.654 ***	3548	0.893	0.029				
Constrained model	M6b	7513.664 ***	3677	0.892	0.028	M6b and M6a	153.010	129	0.001

** *p* < 0.01, *** *p* < 0.001.

## Appendix A

### Perceived Paternal/Maternal Concerted Cultivation Scale (PCC and MCC):

Paternal/Maternal academic involvement

1. My father/mother is nervous about my academic result.
2. My father/mother tries with tremendous effort to improve my academic result.
3. My father/mother frequently consults teachers on my academic performance.
4. My father/mother involves intensively in preparing me for examination.
5. My school report is my father’s/mother’s performance record.

Paternal/Maternal provision of structured activities

1. My father/mother fills out my schedule with numerous activities.
2. I live under my father’s/mother’s schedule.
3. My father/mother involves a lot in preparing my activities.
4. My father/mother gives me less space and time for my own hobbies.
5. My father/mother requires me to attend tutorials or skill-enhancement activities during school holidays.
